# Roles of Staphylococcus aureus Mnh1 and Mnh2 Antiporters in Salt Tolerance, Alkali Tolerance, and Pathogenesis

**DOI:** 10.1128/JB.00611-17

**Published:** 2018-02-07

**Authors:** Manisha Vaish, Alexa Price-Whelan, Tamara Reyes-Robles, Jun Liu, Amyeo Jereen, Stephanie Christie, Francis Alonzo, Meredith A. Benson, Victor J. Torres, Terry A. Krulwich

**Affiliations:** a Department of Pharmacological Sciences, Icahn School of Medicine at Mount Sinai, New York, New York, USA; b Department of Microbiology, NYU Langone Medical Center, New York, New York, USA; University of Chicago

**Keywords:** multisubunit cation/proton antiporter I, Staphylococcus aureus, Mnh1, Mnh2, pathogenesis

## Abstract

Staphylococcus aureus has three types of cation/proton antiporters. The type 3 family includes two multisubunit Na^+^/H^+^ (Mnh) antiporters, Mnh1 and Mnh2. These antiporters are clusters of seven hydrophobic membrane-bound protein subunits. Mnh antiporters play important roles in maintaining cytoplasmic pH in prokaryotes, enabling their survival under extreme environmental stress. In this study, we investigated the physiological roles and catalytic properties of Mnh1 and Mnh2 in S. aureus. Both Mnh1 and Mnh2 were cloned separately into a pGEM3Z+ vector in the antiporter-deficient KNabc Escherichia coli strain. The catalytic properties of the antiporters were measured in everted (inside out) vesicles. The Mnh1 antiporter exhibited a significant exchange of Na^+^/H^+^ cations at pH 7.5. Mnh2 showed a significant exchange of both Na^+^/H^+^ and K^+^/H^+^ cations, especially at pH 8.5. Under elevated salt conditions, deletion of the *mnhA1* gene resulted in a significant reduction in the growth rate of S. aureus in the range of pH 7.5 to 9. Deletion of *mnhA2* had similar effects but mainly in the range of pH 8.5 to 9.5. Double deletion of *mnhA1* and *mnhA2* led to a severe reduction in the S. aureus growth rate mainly at pH values above 8.5. The effects of functional losses of both antiporters in S. aureus were also assessed via their support of virulence in a mouse *in vivo* infection model. Deletion of the *mnhA1* gene led to a major loss of S. aureus virulence in mice, while deletion of *mnh2* led to no change in virulence.

**IMPORTANCE** This study focuses on the catalytic properties and physiological roles of Mnh1 and Mnh2 cation/proton antiporters in S. aureus and their contributions under different stress conditions. The Mnh1 antiporter was found to have catalytic activity for Na^+^/H^+^ antiport, and it plays a significant role in maintaining halotolerance at pH 7.5 while the Mnh2 antiporter has catalytic antiporter activities for Na^+^/H^+^ and K^+^/H^+^ that have roles in both osmotolerance and halotolerance in S. aureus. Study of S. aureus with a single deletion of either *mnhA1* or *mnhA2* was assessed in an infection model of mice. The result shows that *mnhA1*, but not *mnhA2*, plays a major role in S. aureus virulence.

## INTRODUCTION

Staphylococcus aureus is a commensal colonizer of 20 to 30% of healthy people as normal flora of the nasopharynx, skin, and other secondary niches (1). Colonization leads to elevated risk for metastatic S. aureus infections of the host. S. aureus strains cause a large diversity of infections (1, 2). Factors underpinning this diversity include robust stress resilience, biofilm formation, resistance to successive antibiotics, generation of antibiotic-tolerant persisters, and evasion of the host immune response (3–6). S. aureus not only is prevalent in hospital settings but also has survival efficiency in hostile environments. It successfully survives in high saline up to 25% NaCl, which is commonly found in canned foods. The low water activity (a_w_) makes the bacteria uniquely resistant to drying and capable of growing and producing enterotoxins in foods with low a_w_ (7, 8). S. aureus lives in highly alkaline (pH up to 9.5) conditions that are found in garden soil, sewage, ground water, and some parts of the human gut (9, 10). Its ability to grow under osmotic and pH stress underpins the ability of S. aureus to thrive in a wide variety of foods, including cured meats that do not support the growth of other foodborne pathogens (11), and is responsible for staphylococcal food poisoning. Such high tolerance of salt and alkaline pH is largely due to the activity of antiporters found in the plasma membrane, which remove toxic cations from the cytoplasm and enable S. aureus to survive under diverse challenging conditions. S. aureus has three families of antiporters called cation/proton antiporters (CPA), namely, CPA1, CPA2, and CPA3. In the present study, we have focused on two homologous, multisubunit cation/proton antiporters of S. aureus, Mnh1 and Mnh2 of the CPA3 family. Most bacterial cation/proton antiporters are the products of single genes encoding a hydrophobic transporter that often functions as a homodimer (12, 13). In contrast, both S. aureus Mnh antiporters are expressed from two different operons, each of which encodes seven genes whose hydrophobic products are designated MnhA to MnhG. The single homologous operon of Listeria monocytogenes also has seven subunits (14), with more sequence homology to S. aureus Mnh1 than to Mnh2. In contrast, some bacteria, e.g., Vibrio cholerae and Pseudomonas aeruginosa, have a six-protein variation in which the first two genes are fused (15–17). Both of these hetero-oligomeric antiporter types are categorized in the cation/proton antiporter (CPA) family 3 database, Transport DB (18). These are called Mrp antiporters representing multiple resistances and pH roles (18, 19). Mrp-type antiporters were found initially in alkaliphilic Bacillus halodurans (20), but many nonalkaliphiles have Mrp/Mnh antiporters with roles in pH homeostasis, halotolerance, osmotolerance, and resistance to cholate (15, 21–23); P. aeruginosa mrp also supports pathogenesis (16).

Although the Mnh1 antiporter of S. aureus was among the early Mrp-type antiporters described and was shown to catalyze Na^+^/H^+^ antiport activity (21), its roles have not been extensively explored. An extensive study showed that S. aureus strains do not have complex I-type NADH oxidoreductases (24). As detailed structural information became available for a bacterial complex I (25), Mopathi and Hägerhäll (26) showed that the Mnh1 antiporter of S. aureus lacks features required to harness a complex I type of electron transport module. They proposed that Mrp-type antiporters are secondary antiporters that are likely progenitors of protein modules that gained the ability to partner with electron transport modules in other bacteria (27, 28). The elevated membrane potential that accompanies activity of an Mnh antiporter is explained by feedback loops. High pH or elevated cation levels increase the demand for proton motive force (PMF) to support the increased cation/proton antiport activity required for pH and/or cation homeostasis. In such instances, increased expression of electron feeders of respiratory chain and/or respiratory components themselves have been observed (29, 30). Finally, a purified Mrp homologue of Mnh antiporters was co-reconstituted in proteoliposomes with an ATPase to establish a PMF and was demonstrated to be functional with no oxidoreductase activity involved (31). The Mnh1 antiporter is thus expected to function as a secondary antiporter that catalyzes PMF-dependent Na^+^/H^+^ antiport activity as originally suggested (21). S. aureus strains also have, as already noted, a second Mnh that is designated Mnh2 (17). In this study, the catalytic capacities and physiological roles of the two Mnh antiporters of S. aureus were examined in two S. aureus strains, SH1000 and Newman. Using S. aureus Newman, effects of functional loss of Mnh1 or Mnh2 were also assessed in a murine infection model.

## RESULTS

### Expression of Mnh1 is largely constitutive while Mnh2 is induced by σ^B^.

The two *mnh* operons of S. aureus are transcribed in different directions from different loci in the chromosomes. Additionally, while the *mnh1* operon consists solely of the seven *mnh1* genes, an integrase-recombinase gene (*itr*) was found to precede the seven *mnh2* genes. The S. aureus chromosome encodes six other candidate cation/proton antiporters from three additional antiporter families (CPA1, CPA2, and NhaC). The single CPA2 family member was reported to be a receptor for signaling nucleotide c-di-AMP (32).

A survey of microarray-based gene expression experiments indicated that *mnh1* genes are relatively unresponsive to environmental changes (33) (Table 1). In contrast, the *mnh2* operon is controlled by σ^*B*^ and is upregulated under stressful conditions, with patterns that often diverge from expression patterns of *mnh1* (34, 35).

**TABLE 1:** Effects of selected conditions on expression of *mnh1* and *mnh2* genes, compiled from the Staphylococcus aureus Transcriptome Meta-Database (SATMD)

Gene	Relative expression by condition (reference)								
	Cold shock (59)	Stringent response (59)	Berberine chloride (60)	Alkali (61)^*a*^	Inorganic acid (61)^*b*^	2 M NaCl (41)	SigB (35)	Fusidic acid (62)	Biofilm vs planktonic (63)^*c*^
*mnhA1*		↓	↓	↓					
*mnhB1*		↓	↓						
*mnhC1*		↓	↓	↓					
*mnhD1*		↓							
*mnhE1*	↓	↓		↓		↓			
*mnhF1*		↓		↓		↓			
*mnhG1*		↓		↓		↓			
*mnhA2*	↓		↑	↑			↑		
*mnhB2*							↑	↑	↓
*mnhC2*							↑	↑	↓
*mnhD2*			↑	↑	↓		↑	↑	
*mnhE2*		↓	↑	↑	↓		↑	↑	
*mnhF2*		↓	↑	↑	↓		↑	↑	
*mnhG2*	↓	↓	↑	↑	↓			↑	

a Alkali, pH 10 with NaOH.

b At pH 4 with HCl.

c At an OD value of 1.0.

**FIG 1:**
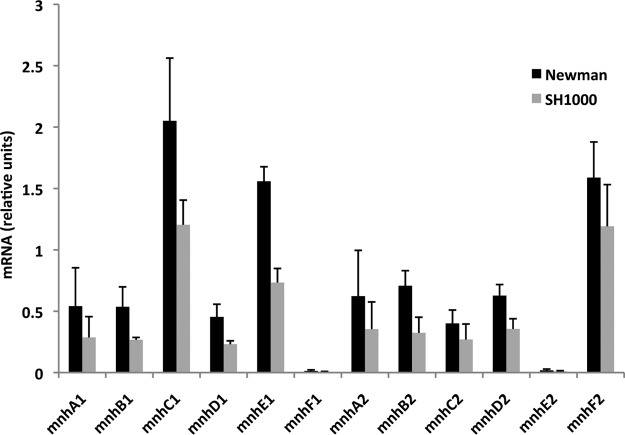
Expression of *mnh1* and *mnh2* operon genes in S. aureus Newman and SH1000 strains grown in defined LB0 medium with no added salt. Data represent the average of biological triplicates. Error bars represent standard deviations. *pyk* and *fabD* were used as a reference genes.

We compared expression of *mnh1* and *mnh2* operon genes in Newman and SH1000 grown in Luria-Bertani broth (LB) without added NaCl or KCl (LB0 medium) until log phase. Expression levels of the *mnh1* and *mnh2* operon genes were higher in Newman than in SH1000 (Fig. 1). These data sets were analyzed using a quantitative PCR (qPCR) assay on each of the two strains.

### Comparison of catalytic properties and physiological roles of the two Mnh antiporters.

**FIG 2:**
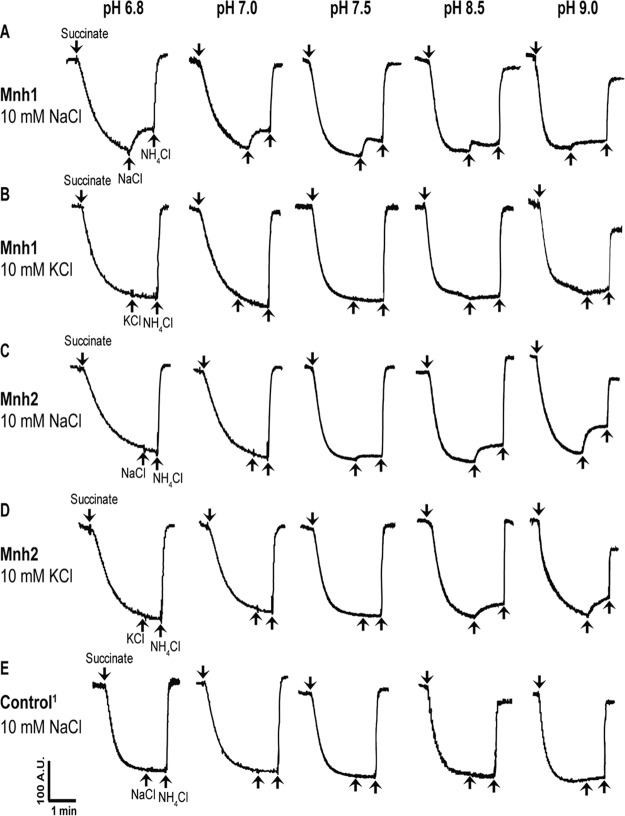
Antiport activity of Mnh1 and Mnh2. Mnh1 (A and B) and Mnh2 (C and D) were assayed for Na^+^/H^+^ and K^+^/H^+^ antiport activity, as a function of pH. (E) An empty vector control set of Na^+^/H^+^ antiport activity assays was also conducted as a function of pH. Membrane vesicles from pregrown cells were prepared in an inside out orientation relative to the cell membrane from E. coli KNabc cells expressing S. aureus SH1000 *mnh1* or *mnh2* in pGEM3zf+ vector as described earlier (57). Succinate (2.5 mM) addition initiated PMF generation, as monitored via the quenching of fluorescence of acridine orange, a ΔpH probe present at 1 μM. Antiport activity was assessed from the percent dequenching of acridine orange fluorescence after addition of 10 mM NaCl or KCl. Addition of NH_4_Cl to each assay abolished residual ΔpH and established a baseline. The tracings shown are representative of assays that were carried out on at least three independent vesicle preparations, with the assays conducted in duplicate for each preparation. A.U., arbitrary units. An empty-vector control set of K^+^/H^+^ antiporter assays was also conducted and resulted in no dequenching with KCl addition (data not shown).

Assays for Mnh antiport activity were conducted in everted (inside out) membrane vesicles of Escherichia coli KNabc, a cation/proton antiporter-deficient strain (Table 2). E. coli KNabc was transformed with either an empty control plasmid or a plasmid expressing either the S. aureus SH1000 *mnh1* or *mnh2* operon; raw antiporter data are shown in Fig. 2. Mnh1 catalyzed Na^+^/H^+^ antiport activity at a pH optimum of 7.5 but still exhibited modest antiport activity at pH 9.0. In contrast, Mnh2 catalyzed Na^+^/H^+^ and K^+^/H^+^ antiport activity, showing little Na^+^/H^+^ antiport and no K^+^/H^+^ antiport activity at pH 7.5. As the pH was increased to pH 9.0, increasing Mnh2 antiport activity was observed (Fig. 2), but although not shown, the antiport activity of Mnh2 at pH 9.5 dropped to zero. The profile shown in Table 2 includes the *K_m_* for the antiport activities at the optimum pH for each of the two antiporters, pH 7.5 for Mnh1 and pH 9.0 for Mnh2, in the E. coli host used in the assay. The assays shown in Table 3 indicate that if sufficient [Na^+^] is provided for Mnh2, this antiporter exhibits Na^+^/H^+^ antiport activity comparable to that of Mnh1 at pH 7.5, with either succinate or ATP establishing the PMF that energizes the exchange. Cytoplasmic concentrations of K^+^ in the range of 900 mM are found in S. aureus (36), and this is sufficient to enable Mnh2 to carry out K^+^/H^+^ antiport activity even when the antiporter is functioning at a pH significantly below its optimal pH range (Table 3). At such a high concentration of potassium salt in the cytoplasm, the Mnh1 antiporter was inactive, which shows that Mnh2 plays a more important role in osmotolerance. In this study, we report Mnh2 antiport activity for the first time.

**TABLE 2:** Activity profile of Mnh1 and Mnh2^*a*^

Antiporter and substrate (15 mM)	Assay pH^*b*^	Antiport activity (% dequenching)	*K_m_* (mM)^*c*^
Mnh1: Na^+^	7.5	21 ± 1	0.56 ± 0.02
Mnh2			
Na^+^	9.0	36 ± 1	0.30 ± 0.01
K^+^	9.0	39 ± 1	0.39 ± 0.21

a Antiporter assays used 2.5 mM Tris-succinate as the electron donor to the respiratory chain so that a proton motive force, acidic inside, was generated across the everted membrane vesicles. The vesicles were prepared from antiporter-deficient E. coli KNabc transformed with a pGEM3zf+ vector into which either *mnh1* or *mnh2* was cloned along with a vector control.

b The antiporter assays were conducted at pH values from pH 7 to pH 9.5. The assay pH that is shown was the pH that yielded the highest activity.

c The *K_m_* (±standard deviation) was calculated at the assay pH shown and is the average from three independent experiments that were conducted with duplicate assays with freshly prepared vesicles prepared from pregrown cells as described in Materials and Methods.

**TABLE 3:** Mnh1 and Mnh2 antiport activities at pH 7.5^*a*^

Antiporter and substrate	Concn (mM)	Antiport activity (% dequenching)^*b*^	
		Succinate	ATP
Mnh1: Na^+^	15	21 ± 1	24 ± 1
Mnh2			
Na^+^	600	27 ± 2	28 ± 1
K^+^	900	28 ± 3	37 ± 1

a Antiporter assays were conducted at pH 7.5 using the concentrations of Na^+^ or K^+^ indicated, which were determined in preliminary experiments not shown. The efficacies of Tris-succinate and Tris-ATP in providing energy for proton motive force (PMF) generation were compared in each of the duplicate assays conducted in three independent experiments using freshly prepared vesicles from cells that were pregrown as described in Materials and Methods.

b Succinate generates PMF by donating electrons to the electron transport chain of the antiporter-deficient E. coli KNabc host, while ATP generates PMF by powering the E. coli KNabc F_1_F_o_-ATPase, which directly pumps protons inward in the everted membrane vesicle system.

**FIG 3:**
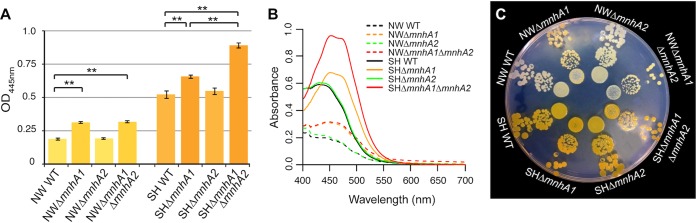
Carotenoid changes observed in single and double mutants of S. aureus SH1000 and Newman relative to levels in the wild type. (A) Carotenoid measurements for S. aureus SH1000 and Newman wild-type, Δ*mnhA1*, Δ*mnh2*, and Δ*mnh1* Δ*mnh2* strains. (B) Sample absorbance spectra for methanol extracts of S. aureus SH1000 and Newman wild-type and Δ*mnhA1*, Δ*mnhA2*, and Δ*mnhA1* Δ*mnhA2* strains. (C) Radial plating of S. aureus SH1000 and Newman for carotenoid comparison between wild-type and mutant strains. **, *P* < 0.01 by unpaired *t* tests between results for the wild-type and Δ*mnhA1* strains and between results for the wild-type and Δ*mnhA1* Δ*mnhA2* strains.

In order to compare the major physiological contributions of Mnh1 and Mnh2, Δ*mnhA1* and Δ*mnhA2* single deletion mutants and a double mutant with deletions in both Δ*mnhA1* and Δ*mnhA2* were constructed in S. aureus SH1000 and Newman. SH1000 had been used in the work that led to the proposal that Mnh was part of a PMF-generating NADH dehydrogenase (37). Deletion of the *mnhA* gene in SH1000 and Newman completely inactivated Mnh1 and Mnh2, respectively. It was noted that the Δ*mnhA1* mutant formed smaller colonies that were hyperpigmented and more orange than the colonies of the staphyloxanthin-containing wild-type strain. A Δ*mnh1* and Δ*mnh2* double mutant showed significantly more pigmentation than a Δ*mnh1* single deletion in SH1000. There was not much difference in the pigment levels of the Δ*mnh1* and Δ*mnh1 Δmnh2* strains in Newman (Fig. 3A). The increase in pigment is recognized as a stress response (38, 39). All the strains of SH1000, mutants and wild type, showed more pigmentation than Newman strains (Fig. 3C). The reason for more pigmentation in the SH1000 strain is overexpression of the *sigB* genes, which also regulates the stress responses. The peaks in absorbance spectra of methanol extracts of the mutant showed a slight shift relative to wild-type spectra (Fig. 3B), suggesting the presence of additional carotenoids that may include staphyloxanthin intermediates (40). No similar carotenoid elevation or shift was observed in the Δ*mnh2* mutant.

To further explore the roles of the two Mnh antiporters in S. aureus physiology, we studied these antiporters in the commonly used methicillin-susceptible S. aureus strains Newman and SH1000. Growth experiments were conducted in LB0 medium, with no addition of sodium or potassium to the base medium (LB0). As determined by flame photometry, the sodium concentration in LB0 medium was 14.1 mM, and the potassium concentration was 7.3 mM (41).

**FIG 4:**
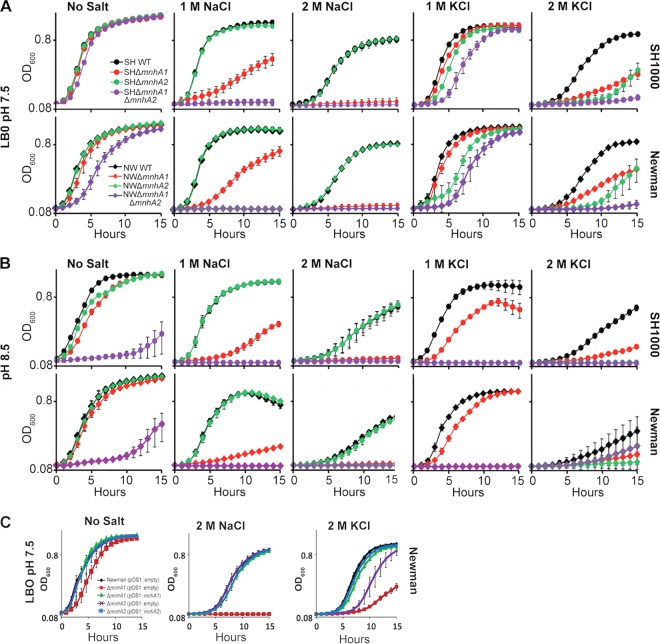
(A) Effects of NaCl and KCl on growth of S. aureus SH1000 (SH) and Newman (NW) wild-type (WT), Δ*mnhA1* and Δ*mnhA2* mutants, and the Δ*mnhA1* Δ*mnhA2* double mutant. For the experiments shown in all panels, strains were grown in LB0 medium at 37°C, pH 7.5. Additions of 1 M, 2 M NaCl, and 1 M, 2 M KCl were made, as indicated. (B) The same strains were grown as described for panel A except that the pH was 8.5. (C) For the complementation experiments, the Newman Δ*mnhA1* mutant was transformed with the pOS1 vector containing the *mnhA1* gene while the Δ*mnhA2* strain was transformed with pOS1 containing *mnhA2*. For the complementation controls, pOS1 with no insert (pOS1::empty) was transformed into each of the three strains (i.e., wild-type, Δ*mnhA1*, and Δ*mnhA2* strains). The growth curves for this and other experiments are the average of three independent experiments in which duplicate assays were conducted; the error bars represent the standard deviations.

None of the mutants, not even the Δ*mnhA1 ΔmnhA2* mutant, showed a growth defect in LB0 medium at pH 7.5 without added salt, whereas addition of 1 M NaCl resulted in a significant growth defect in the Δ*mnhA1* strain and no growth for the double mutant (Fig. 4). Increased pH exacerbated the growth defects both with and without added sodium. No obvious role for the Mnh2 antiporter was observed in coping with addition of 1 M NaCl at pH 7.5 or 8.5 except that the double mutant exhibited a growth deficit relative to the growth of the Δ*mnhA1* single mutant at pH 8.5. When 1 M KCl was added instead, no significant defect was observed in the Δ*mnhA2* mutant at pH 7.5, but at pH 8.5 there was a significant growth deficit, with the single mutant being almost as impaired as the double mutant. In general, the Δ*mnhA1* mutant exhibited less growth inhibition in response to added 1 M KCl than to 1 M NaCl, and the Δ*mnhA2* mutant exhibited less growth inhibition in response to added 1 M NaCl than to 1 M KCl. The results were consistent with distinct but partially overlapping roles of the two Mnh antiporters. The Δ*mnhA1*, Δ*mnhA2*, and Δ*mnhA1 ΔmnhA2* mutant strains were more sensitive to salt and pH stress in the Newman than in the SH1000 background.

The growth patterns of wild-type S. aureus Newman were compared to those of mutants lacking Mnh1 or Mnh2 activity (Δ*mnhA1* or Δ*mnhA2* strain). Even in the absence of added Na^+^, there was some growth inhibition of the Δ*mnhA1* mutant strain but not of the Δ*mnhA2* mutant strain (Fig. 4). In the presence of 2 M NaCl at pH 7.5, the Δ*mnhA1* mutant exhibited no growth whereas growth of the Δ*mnhA2* strain was unaffected. In the presence of 2 M KCl at pH 7.5, both the Δ*mnhA1* and the Δ*mnhA2* mutants showed significant growth defects, with the Δ*mnhA1* mutant exhibiting a more severe defect than the Δ*mnhA2* mutant. Plasmid-based complementation restored the growth of these mutants to the wild-type level under the conditions tested (Fig. 4C).

**FIG 5:**
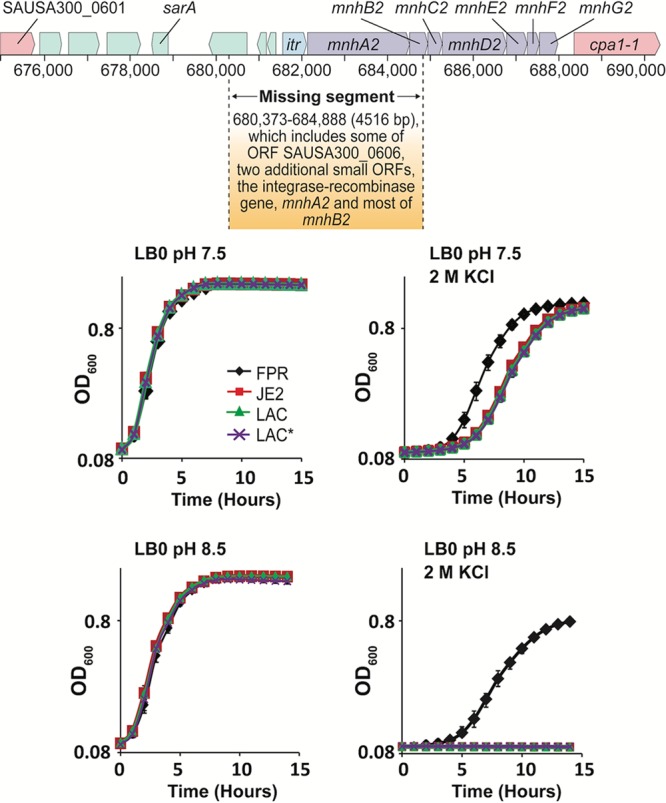
A partial *mnh2* deletion in S. aureus JE2 and two related strains leads to a growth defect at high osmolarity that is exacerbated by elevated pH. (Top) The JE2 strain and related LAC and LAC* strains were found by sequencing to have a partial *mnh2* deletion that occurred upon a transposition from another chromosomal region. The resulting deletion that includes part of the *mnh2* locus extends upstream beyond *mnh2* to several more small ORFs. This is shown diagrammatically using the numbering of S. aureus USA300 *_*FPR3757 so that the numbers up to the displacement correspond to wild-type numbering, without the effect of the deletion from the other segment that relocated. The deletion affecting *mnh2* is shown by the missing segment described in the box. (Bottom) Growth curves comparing JE2, LAC, and LAC* to growth of the reference strain, USA300_FPR3757, with and without the addition of 2 M KCl at either pH 7.5 or 8.5. The growth curves are the average of three independent experiments, with the error bars representing the standard deviations.

All efforts to make a clean deletion of the *mnhA1* gene in naturally occurring Δ*mnh2* strains like JE2, LAC, and LAC* failed. However, when we tried to disrupt the *mnhA1* gene by introducing a chloramphenicol resistance cassette in Δ*mnhA2* Newman, we successfully obtained a double deletion of the Δ*mnh1* and Δ*mnh2* genes. JE2, LAC, and LAC* strains showed a phenotype similar to that of the SH1000 and Newman Δ*mnhA2* strains (Fig. 5).

### A transposition that disrupts the *mnh2* operon is found in the S. aureus USA300 LAC strains in which Mnh1 was initially suggested to be essential.

Fey et al. (42) reported that the *mnh*1 genes are candidates for essential genes, based on a screen that was conducted with community-acquired methicillin-resistant S. aureus (CA-MRSA) USA300_LAC derivatives, with the JE2 strain used for transposon mutagenesis. The USA300_FPR3757 genomic sequence (NCBI reference sequence NC_007793) was used as the reference sequence. A critical observation was the absence of some *mnh2* genes, along with that of the *itr* gene of the *mhh2* operon and a few small unrelated genes that are usually upstream of the operon. Sequence examinations revealed that these genes were replaced during transposition of an ∼13.1-kb element from another region of the genome into the region containing the *mnh2* operon. The transposition resembled but was not completely identical to a transposition reported by Highlander et al. (43) in S. aureus USA300-HOU-MR. We found similar insertions in additional S. aureus strains, with an overall frequency below 5% in the 67 strains that were examined on the NCBI site. The deletion site is shown in Fig. 5 using the framework of the USA300_FPR3757 reference strain, which has intact *mnh1* and *mnh2* operons so that the sequence numbers retain the wild-type numbering rather than reflect the downstream loss of a transposed element. Growth curves are shown for the reference wild-type strain, S. aureus FPR3757, and three other related strains made for various screens. JE2 is the strain into which the deletions were made in the study of Fey et al. (42), LAC is the parental strain, and LAC* is an erythromycin-sensitive version of LAC. All three strains exhibited sensitivity to 2 M KCl that was exacerbated by elevation of the pH from 7.5 to 8.5 (Fig. 4). This was consistent with loss of Mnh2 function. Multiple attempts to inactivate the intact *mnh1* locus in the JE2 and LAC strains were unsuccessful, in contrast to our success in making a double mutant in S. aureus SH1000 and Newman. In the JE2 and LAC strains it is possible that Mnh1 is essential. The viability of the double mutant of Newman shows that *mnh1* is not essential. The growth patterns of JE2, LAC, and LAC* were similar to the growth of the Δ*mnh2* mutant strain of methicillin-sensitive Newman and SH1000.

### Mnh1 is required for fitness and pathogenesis *in vivo*.

**FIG 6:**
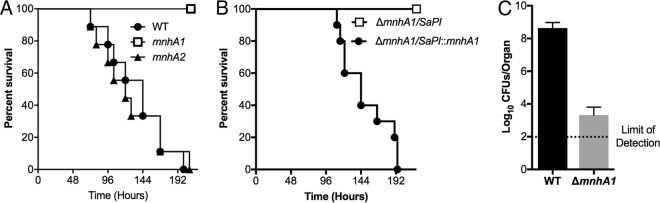
Mnh1 is required for S. aureus fitness and pathogenesis *in vivo*. (A) Survival of mice infected intravenously with ∼1 × 10^7^ CFU of the isogenic S. aureus wild-type (*n* = 10), Δ*mnhA1* (*n* = 10), or Δ*mnhA2* (*n* = 10) strain. (B) Survival of mice infected intravenously with 2 × 10^7^ to 4 × 10^7^ CFU of the isogenic S. aureus Δ*mnhA1*/SAPI (2.2 × 10^7^ CFU; *n* = 10) or Δ*mnhA1*/SAPI::*mnhA1* (3.45 × 10^7^ CFU; *n* = 10) strain. (C) CFU counts from kidneys isolated at the end of the experiment from mice infected with the Δ*mnhA1* strain compared to CFU counts from kidneys isolated at ∼96 h postinfection with the wild-type strain. The bar indicates the median. The dotted line indicates the limit of detection.

To evaluate the contributions of Mnh1 and Mnh2 to S. aureus pathogenesis, mice were infected systematically with isogenic mutants of strain Newman lacking a functional form of either Mnh1 or Mnh2 and compared to a wild-type control with respect to their virulence. The strain with the Δ*mnhA2* deletion exhibited virulence similar to that of the wild-type strain, whereas the strain with the Δ*mnhA1* deletion showed marked attenuation of virulence (Fig. 6A). To ensure that the phenotype observed was only dependent on the lack of a functional Mnh1 antiporter, a complemented strain was generated by introducing a wild-type copy of *mnhA1* into the Δ*mnhA1* deletion mutant as a single copy inserted into the SaPI1 site in the chromosome, as had been done previously (44). Expression of the chromosomal wild-type copy of *mnhA1* reversed the virulence defect of the strain with the Δ*mnhA1* deletion (Fig. 6B). Consistent with the survival data, the Δ*mnhA1* deletion mutant exhibited an ∼5-log reduction in bacterial burden in the kidneys of infected mice relative to the level with wild-type Newman (Fig. 6C).

## DISCUSSION

This study confirms that both Mnh1 and Mnh2 of S. aureus are secondary antiporters that catalyze K^+^ and/or Na^+^ ion efflux in exchange for H^+^ ions. The PMF provides the required energy just as well when proton pumping is energized by ATPase activity as when ion pumping via the electron transport chain is involved (Table 3). S. aureus strains are usually able to grow in a range of pHs from pH 5 to at least pH 9 (45) and persist at high osmolarity (41). Mnh1 and Mnh2 have important roles in halotolerance and osmotolerance, respectively (Fig. 4). Nonetheless, they have enough functional overlap that each Mnh can play a critical compensating role in viability when the other Mnh has lost function. Inactivation of Mnh1 by deletion of its first structural gene (*mnhA1*) resulted in a large reduction in the lethal effects of the wild-type strain in a murine systemic infection mode. Lethality was restored by restoration of a functional *mnhA1* into the chromosome (Fig. 6). A comparable deletion of *mnh2* in S. aureus Newman did not result in a virulence change. Thus, Mnh1 but not Mnh2 is of interest as a possible new target for incapacitating S. aureus in animal hosts. The Δ*mnh1* mutant strain is also sensitive to sucrose-generated stress that indicates the osmotolerance of Mnh1 at pH 7.5 to 8.5 (data not shown). Mnh2 would need a high concentration of cytoplasmic K^+^ in order to carry out K^+^/H^+^ antiport activity in the nearly neutral range of pH that is optimal for Mnh1 (Table 2); this contingency is likely to be the rationale for the high cytoplasmic K^+^ levels that have been found in S. aureus strains (36). Mnh2, which supports osmotolerance, may be a target to consider in the context of packaged-food poisoning by S. aureus strains.

Several screens, with different methodologies (46–50), have been carried out to identify genes of diverse S. aureus strains that are essential for fitness in particular settings. The screens with highly stress-resistant S. aureus strains SH1000 and Newman have not shown an essential role for Mnh antiporter genes (47). Fey et al. (42) noted that Mrp antiporter genes of the single Bacillus subtilis Mrp-type antiporter are likely to be essential as they were among the genes that lacked transposon (Tn) insertions in a similar screen. However, we were able to get viable strains with a double deletion of Δ*mnh1* and Δ*mnh2* in the Newman strain that suggests that there is leeway. Similarly, a B. subtilis mutant with a full Δ*mrpA-mrpG* deletion and strains with individual deletions of the B. subtilis mrp genes have already been shown to be viable if the mutants are grown at nearly neutral pH in medium without added sodium ions (19, 51).

In the Pfam database of protein families, Mnh antiporters had, until recently, been grouped together with complex I-type proton pumps under Pfam00361 and identified as oxidored-q1m. Recently, the Pfam curators introduced a distinct Pfam00361 designation, proton_antipo_M, to accommodate Mrp-type secondary antiporters, including Mnh1 and Mnh2, which are independent antiporters that do not carry out redox reactions (52). Another point of confusion had been caused by inconsistent name assignments for the *mnh1* and *mnh2* antiporters in S. aureus genome annotations. A significant number of *mnh2* antiporters are annotated as *mnh1* antiporters and vice versa. This should now be easy to correct since the *mnh1* operon has only the seven structural genes, and the *mnh2* operon can thus be distinguished by its additional integrase-recombinase gene, *itr*, which precedes the *mnh2* structural genes.

## MATERIALS AND METHODS

### Bacterial strains and their cultures, plasmids, and primers.

The bacterial strains, plasmids, and primers used in this study are listed in Tables 4 and 5.

**TABLE 4:** Bacterial strains and plasmids used in this study

Strain or plasmid	Description	Source or reference
Strains		
Staphylococcus aureus		
SH1000	S. aureus 8325-4 with repaired *rsbU*	64
SH1000 Δ*mnhA1*	Markerless deletion of the *mnhA1* gene in SH1000 (locus tag SAOUHSC_00889)	This study
SH1000 Δ*mnhA2*	Markerless deletion of the *mnhA2* gene in SH1000 (locus tag SAOUHSC_00625)	This study
SH1000 Δ*mnhA1* Δ*mnhA2*	Markerless deletion of the *mnhA1* and *mnhA2* gene in SH1000	This study
Newman	Wild type (clinical isolate)	65
Newman Δ*mnhA* (VJT39.95)	Markerless deletion of the *mnhA1* gene in Newman (locus tag NWMN_0822)	This study
Newman Δ*mnhA2*	Markerless deletion of the *mnhA2* gene in Newman (locus tag NWMN_0593)	This study
FPR3757	Wild type (USA300)	66
JE2	USA300 (derivative of LAC)	42
LAC	Wild type (USA300)	67
LAC*	USA300 (derivative of LAC)	68
RN4220	Restriction-deficient intermediate strain	69
RN9011	RN4220/pRN7023 (SaPI1 integrase, *cat194*)	44
Escherichia coli		
KNabc	Δ*nhaA* Δ*nhaB* Δ*chaA* (derived from E. coli TG1	70
DH5α	Transformation strain	71
DH5α-T1^R^	Competent cells for site-directed mutagenesis	Invitrogen
Plasmids		
pMAD	*E*. coli/S. aureus shuttle vector	53
pOS1	S. aureus shuttle vector	72
pGEM-3Zf(+)	Derivative of the pGEM-3Z vector	Promega
pGEMMnh	pGEM-3Zf(+) containing the *mnh1* operon with putative promoter	30
pAMPW15	pGEM-3Zf(+) containing the *mnh2* operon with putative promoter	This study
pJC1112	SaPI1 attS suicide vector, erythromycin resistant (*ermC*)	73
pRN7023	SaPI1 integrase vector, chloramphenicol (*cat194*)	73

**TABLE 5:** Primers used in this study

Function (strain) and primer name^*a*^	Sequence
*mnhA1* deletion (SH1000)	
mnhA1 del 1-1	ATAGGTACCACGCGTATTGGTCCATGTTT
mnhA1 del 1-2	TGAGTTTGTTACATATTGCGGTGGTGCTTGGTATCGCAGGATT
mnhA1 del 2-1	AATCCTGCGATACCAAGCACCACCGCAATATGTAA
mnhA1 del 2-2	ATAGGATCCCAGCCACCATCTGCAAGTT
*mnhA2* deletion (SH1000)	
mnhA2 del 1-1	ATAGGATCCTGTTTCTCCAGTAAGTCGCTCA
mnhA2 del 1-2	GCTCTAAAGTCACCAAGTATCGCCGCTATGTACCCGGCATATT
mnhA2 del 2-1	AATATGCCGGGTACATAGCGGCGATACTTGGTGACTTTAGAGC
mnhA2 del 2-2	ATAGAATTCGACGGTGACGCTGAACATAA
*mnhA1* deletion (Newman)	
mnh1-F1369	ATAGGATCCCCACTTTGATTTCACCTTGTTG
mnhA1 del 2-1	AATCCTGCGATACCAAGCACCACCGCAATATGTAACAAACTCA
mnh1-R4986	ATAACGCGTCAGTACCATGTTTAAGTCCGCCC
mnhA1 del 1-2	TGAGTTTGTTACATATTGCGGTGGTGCTTGGTATCGCAGGATT
*mnhA2* deletion (Newman)	
mnh2-F1473	ATAGGATCCGAAGCGCTCAAGATAAAATCTG
mnhA2 del 1-2	GCTCTAAAGTCACCAAGTATCGCCGCTATGTACCC
mnh2-R4960	ATAGAATTCCCATACGTTCCCATACTCATAA
mnhA2 del 2-1	AATATGCCGGGTACATAGCGGCGATACTTGGTGACTTTAGAGC
*mnhA1* deletion with CAT^*b*^ gene (Newman)	
mnhA1.UpF_BamH1	ATATAGGATCCGTAGCTATGGTGTCACGA
mnhA1.UpR_Xho1	ATTTTCGCTCGAGTTAGAGTGCGAATATTAACGG
CatF_Xho1	ATATACTCGAGCGAAAATTGGATAAAGTGGG
CatR_kpn1	ATATAGGTACCGTACAGTCGGCATTATCTCA
mnhA1.DnF_kpn1	ACTGTACGGTACCATCGCAGGATTAGCTGTA
mnhA1.DnR_EcoR1	ATATAGAATTCGACACTAATTGCTGTGAG
qPCR on *mnh1* (SH1000)	
mnhA1 f	GCACCCGACTTAGCATTGAC
mnhA1 r	ATGACGGACAAACCAACACC
mnhB1 f	TATACACCTGGTGGCGGTTT
mnhB1 r	CGATAGGCGTCGCAATACA
mnhC1 f	GGCGGACTTAAACATGGTACTG
mnhC1 r	TAAGTGCTTGCGGGATAGGA
mnhD1 f	CCTTCTTCGTAATGGGTGTAGC
mnhD1 r	TCATGCGCTGAAAGGTTAGC
mnhE1 f	GCAGTGTTTTGGTTGTTTGTGAC
mnhE1 r	AACACTCTGTGTAAGAGGTAAACAAGAACTA
mnhF1 f	CATTAGATGCGATTGGTCTTCA
mnhF1 r	TGTTCAATCACCTTACCTTTGTCC
qPCR on *mnh2* (SH1000)	
mnhA2 f	GCGGATATGCTCAACACCAA
mnhA2 r	TCCCATGAAAAGCGCACA
mnhB2 f	CGTGTTAAGAACGGTCACGAA
mnhB2 r	CAATAAACCCACCACCAGGA
mnhC2 f	GGTTTGGGATGACTGCGTTT
mnhC2 r	CCCTTAGGCCTTCAATTTCATC
mnhD2 f	GTCATCGGCGCTATAGGTGT
mnhD2 r	CCTGCAAACGTGTTTGTTCC
mnhE2 f	ACAGGTTTTTCAGCGATGATTT
mnhE2 r	TCCAGGGTTCATATCTTTTGTTT
mnhF2 f	TCAAGGGACCTACAACAGCA
mnhF2 r	GAAACGGTGCCCATAAGT

a f, forward; r, reverse.

b CAT, chloramphenicol acetyltransferase.

### Growth conditions.

S. aureus strains were routinely grown in a modified version of Luria-Bertani broth (LB), designated LB0, which is LB without added NaCl or KCl. Cultures were incubated at 37°C with shaking at 225 rpm. For plasmid selection in S. aureus strains, erythromycin was added to the medium at 2.5 μg/ml, and chloramphenicol was added at 10 μg/ml. For growth experiments, a BioTek PowerWave plate reader was used. Overnight cultures were inoculated at an initial optical density at 600 nm (OD_600_) of 0.01 in a total of 200 μl of LB0, and 60 mM Bis-Tris propane and salt were added as indicated in the legend to Fig. 4. The cultures were adjusted to the desired pH with HCl and distributed in individual wells of 96-well plates. The plates were incubated with continuous shaking on the low setting at 37°C. The growth curves were conducted in three independent experiments in triplicate repeats.

### Growth experiments.

Glycerol stocks of S. aureus were inoculated in LB0 medium, pH 7.5, with no salt and grown for 16 h at 37°C with shaking at 225 rpm prior to growth experiments. Overnight cultures were normalized to an OD_600_ of 0.2 with unbuffered LB0 medium, which contains no Bis-Tris propane and is not pH adjusted. Ten microliters of precultures at an OD_600_ of 0.2 was passed into 190 μl of corresponding medium in 96-well microplates for a starting OD_600_ of 0.01 for all growth conditions. Microplate lids were then carefully sealed with 1.2- by 40-cm silicone rubber tape and incubated in 37°C with shaking at 225 rpm in a BioTek PowerWave HT microplate spectrophotometer for 24 h. OD_600_ readings were collected every hour. Growth curves are averages of at least three independent experiments done in duplicate repeats.

### Relative quantitation of staphyloxanthin pigment in wild-type S. aureus SH1000 and Newman and Δ*mnh* mutants.

Staphyloxanthin was extracted from S. aureus cultures using methanol, and relative amounts were quantified by absorbance measurements. Cultures were inoculated at an OD_600_ of 0.01 in 50 ml of LB0 medium and incubated in a 250-ml flask at 37°C in the dark. After 48 h of incubation, 1,000 μl of culture was removed for OD_600_ measurements, and 15 ml of culture was centrifuged at 4,500 rpm for 20 min to harvest cells; the pellet was washed twice with sterile water. Excess water was removed, and 1 ml of methanol was added to each cell pellet. The pellets were vortexed to extract the pigment and incubated at 55°C for 20 min in the dark. Pellets were vortexed every 5 min and centrifuged at 4,500 rpm at 4°C after 20 min. Supernatant was collected in a glass tube. The level of carotenoid pigment was estimated quantitatively by measuring the absorbance at a 445-nm wavelength in a Shimadzu UV 1800 spectrophotometer.

### Radial spot plating of S. aureus for carotenoid color comparison between strains.

Overnight cultures were incubated at 37°C in LB0 medium, pH 7.5, with no salt and with shaking at 225 rpm. After 16 h of growth, cultures were normalized to an OD_600_ of 0.2 using LB0 medium, and then 100-fold serial dilutions were made. The following volumes and dilutions were plated on LB0 medium per radial wing from the inner ring for each strain: 5 μl of 10^−2^ dilutions, 13 μl of 10^−4^ dilutions, and 30 μl of 10^−6^ dilutions. The plate was incubated at 37°C for 42 h and left at 4°C for 24 h. After plates were warmed at room temperature, a picture was taken.

### Construction of markerless deletions in S. aureus by allelic replacement.

Deletions of target genes, which were done in frame, were generated using pMAD (53) according to previously published methods (54). Briefly, ∼1-kb PCR products on either side of the sequence to be deleted were generated and fused by gene SOEing (55). The 2-kb product was ligated into pMAD and transformed into E. coli. After plasmid isolation and sequence verification, the construct was moved into S. aureus RN4220 by electroporation. After isolation from RN4220, the construct was electroporated into the target S. aureus strain. The plasmid was recombined into the chromosome by inoculating a liquid culture for 2 h at the permissive temperature (28°C), followed by overnight inoculation at the restrictive temperature (42°C) and plating of dilutions on LB0 agar containing erythromycin. Merodiploid clones (containing the plasmid recombined into the chromosome) were verified by PCR. To resolve the plasmid out of the chromosome and to generate candidate deletion mutants, liquid cultures of merodiploids were incubated at 28°C without selection and transferred by 1:100 dilutions for 7 days before being plated on LB0 agar. Candidate mutants were screened for loss of erythromycin resistance (confirming loss of the plasmid), and PCR and sequencing were used to confirm exclusive presence of the deleted allele.

The Newman Δ*mnhA1* Δ*mnhA2* mutant was not isolated using these methods; instead, a positive-selection method was utilized. The chloramphenicol acetyltransferase gene of 820 kb was amplified from pOS1 and ligated between the PCR products from either side of the *mnhA1* sequence to be deleted using gene SOEing (55). The 3-kb product was ligated into pMAD and transformed into E. coli. After plasmid isolation and sequence verification, the construct was moved into S. aureus RN4220 by electroporation. After isolation from RN4220, the construct was electroporated into the target S. aureus Newman Δ*mnhA2* strain. Subsequent steps were the same as those described above.

### Complementation of markerless deletions.

Plasmids for complementation of the *mnhA1* and *mnhA2* deletion mutants were constructed using the pOS1 vector. For *mnhA1* complementation, an ∼3-kb product was amplified that contained *mnhA2* and the upstream open reading frame (ORF) that is predicted to encode an integrase/recombinase and to be coexpressed with the *mnh2* operon. This product also contains ∼240 bp of sequence upstream of the gene for the putative integrase/recombinase that includes the binding site for σ^B^, which controls *mnh2* expression (35). These products were ligated into pOS1, and the resulting plasmids were transformed into E. coli. After plasmid isolation and sequence verification, the constructs were moved into S. aureus RN4220 by electroporation. After isolation from RN4220, the constructs were electroporated into the appropriate target mutants of S. aureus Newman.

### Preparation of total RNA and cDNA from S. aureus Newman and SH1000 strains and relative quantification of RNA transcripts by qPCR.

RNA was prepared according to a method described previously (56). Cultures were inoculated in 50 ml of LB0 medium and grown up to an OD_600_ of 0.9 at 37°C. Eight milliliters of culture was added to 8 ml of RNA Protect bacteria reagent (Qiagen) in 50-ml sterile tubes and then vortexed immediately for 5 s and incubated at room temperature for 5 min. Cells were harvested by centrifugation (4,700 rpm, 21°C, and 10 min), the supernatant was poured off, and then the tube was inverted on paper towel for 10 s. Pellets were stored at −80°C overnight. The following day, RNA was isolated using an miRNeasy purification kit (Qiagen) for subsequent steps.

cDNA was synthesized using an iScript cDNA synthesis kit (170-8891; Bio-Rad), and each reaction was performed in a final volume using 200 ng of RNA according to the manufacturer's instructions. Relative qPCR was performed in a 384-well plate at the qPCR shared resource facility (Icahn School of Medicine at Mount Sinai, USA). Primers were designed using Primer 3 software. Reactions were set up in a total volume of 10 μl using 5 μl of cDNA (1:50 dilution) and 5 μl of master mix. Thermo cycling conditions were as follows: 2 min at 95°C; 40 cycles of 95°C for 15 s, 55°C for 15 s, and 72°C for 30 s; a final step of 95°C for 15 s, 60°C for 15 s, and 95°C for 15 s. Samples were run in triplicate, and a no-template control and a no-reverse transcriptase control were run to ensure absence of DNA contamination. Data were analyzed using SDS, version 2.2.1, software (Applied Biosystems, USA).

### Antiporter assays.

Antiporter assays were conducted in everted membrane vesicles prepared from transformants of the triple antiporter-deficient E. coli KNabc strain expressing the empty vector, pGEM3zf+, or either the *mnh1* or *mnh2* operon. The everted membrane vesicles are oriented in such a manner that part of the membrane that is exposed outside the bacterial cells comes inside the vesicles. The transformants were grown overnight and then frozen in liquid nitrogen and stored at −80°C. Preparation of vesicles from the E. coli transformants was conducted using a French press as described earlier (57). The vesicles were used immediately after preparation, without being frozen. The assays also followed a protocol used earlier with the same buffer and pH conditions. Acridine orange was the ΔpH probe used, with measurements conducted using an RF-5301 PC Shimadzu spectrofluorophotometer equipped with a stirrer, with excitation at 420 nm and emission at 500 nm (both with a 10-mm slit). When the respiratory chain is energized by succinate, the respiratory chain starts pumping protons inside the vesicles (as vesicles are everted). The initiation of proton motive force generation is indicated for specific experiments.

### Pathogenicity assays. (i) Generation of *mnhA1* chromosomal integration strains and growth curves for determination of their phenotypes.

To generate isogenic *mnhA1* complementation strains, the entire *mnh* locus was amplified from S. aureus Newman genomic DNA using the oligonucleotide pair VJT1276 (5′-ATATAGGATCCTGAAGCTATATCGATTTTCACACAA-3′) and VJT1277 (5′-ATATAGGTACCAACTGCAGCAAATTGCAAAA-3′) and cloned into the integration vector pJC1112 using BamHI and KpnI restriction sites. The plasmid was transformed into E. coli DH5α and ampicillin resistance-selected clones. A positive recombinant clone was electroporated into RN9011 containing plasmid pRN7023 encoding the SaPI1 integrase to promote single-copy chromosomal integration into the S. aureus SaPI1 site (44) and was selected for erythromycin resistance, as described elsewhere (58). The SaPI1 integrated construct was then transduced into strain VJT39.95 (Newman Δ*mnhA1*) using previously published methods (58).

### (ii) Generation of a pJC1112 control strain.

To generate the pJC1112 (empty vector) control strain, purified plasmid from strain VJT9.54 (E. coli DH5α containing pJC1112) was electroporated into RN9011, followed by transduction into strain VJT39.95 (Newman Δ*mnhA1*) as described above.

### (iii) Murine systemic infection.

Animal infections were done according to protocols approved by the New York University School of Medicine Institutional Animal Care and Use Committee. For *in vivo* experiments, 5-week-old female ND4 Swiss Webster mice (Harlan Laboratories) were anesthetized with 250 μl of Avertin (2,2,2-tribromoethanol dissolved in *tert*-amyl-alcohol and diluted to a final concentration of 2.5% [vol/vol] in sterile saline) intraperitoneally. This was followed by retro-orbital injection of 5 × 10^7^ CFU of wild-type S. aureus Newman, isogenic Newman strains with either a single *mnhA1* deletion or *mnhA2* deletion or with an isogenic Newman *mnhA1* deletion strain complemented in single copy with either the integrase-encoding empty vector pJC1112 (Δ*mnhA*1/pJC1112) or pJC1112 with *mnhA1* in the SaPI1 site (Δ*mnhA1*/pJC1112-*mnhA1*). Mice were monitored for acute infection and signs of morbidity, at which points mice were sacrificed, and survival curves were plotted over time.
